# Utility of Specific IgE to Ara h 2 in Italian Allergic and Tolerant Children Sensitized to Peanut

**Published:** 2016-08-09

**Authors:** Pasquale Comberiati, Laura Colavita, Federica Minniti, Giulia Paiola, Carlo Capristo, Cristoforo Incorvaia, Diego Giampiero Peroni

**Affiliations:** 1 *Department of Scienze Chirurgiche, Odontostomatologiche e Materno-infantili, section of Paediatrics, University of Verona, Italy.*; 2 *Department of Paediatrics, Unit of Genetics and Pediatric Immunology, University Hospital ofMessina, Italy.*; 3 *Department of Paediatrics, Faculty of Medicine, Second University of Naples, Naples, Italy.*; 4 *Allergy/Pulmonary Rehabilitation, ICP Hospital, Milan, Italy.*; 5 *Department of Clinical and Experimental Medicine, section of Paediatrics, University of Pisa, Italy.*

**Keywords:** Allergy, Ara h 2, peanut, specific IgE

## Abstract

Emerging data suggest that measurement of serum IgE to peanut components can be clinically helpful and more accurate than IgE to whole peanut to predict peanut allergy. Not all studies have used prospective samples, multiple components and oral challenges. Currently, there are no data on this topic involving Italian children. 32 patients (23 males; median age 9 years) with reported history for peanut allergy and evidence of peanut sensitization (skin prick test to peanut extract ≥ 3mm) have been analyzed for serum IgE to whole peanut and recombinant allergen components Ara h 1, 2, 3, 8, and 9 with Immuno CAP and completed an open oral food challenge with peanut. 12 (37.5%) children had a positive challenge to peanut and were considered allergic. No differences were seen between the median values of IgE to peanut, Ara h 1, 3, 8 and 9 in allergic and tolerant children to peanut challenge. Noteworthy, 5 of 20 tolerant children had IgE to peanut> 15 kUA/l which is commonly considered a predictive value of peanut allergy. Conversely, a significant difference was seen when comparing the median value of IgE to Ara h 2 in the two groups: 0.75 kUA/l (IQR: 0.22-4.34 kUA/l) in allergic children versus 0.1 kUA/l (IQR: 0.1-0.12 kUA/l) in tolerant ones (P< 0.001). IgE levels to Ara h 2 are significantly higher in children that react to oral peanut challenge. Our findings in Italian children have been in line with recent reports in various populations of Northern Europe, the US and Australia and add confirmatory evidence that analysis of IgE to Ara h 2 could reduce the need for peanut challenge in suspected allergic patients.

Food allergy is a big clinical and public health problem in the world both for its frequency (on the rise) and for the risk of life-threatening events, with need of adrenaline prescription. In Europe, estimated lifetime prevalence of food allergy is 17.3% (point prevalence 6%) (1). Peanut is one of the most common (from 0.5 up to 1.8% of allergic children in western countries) and dangerous foods due to IgE-mediated reactions. Unlike other common pediatric food allergies (eggs, milk), the peanut allergy does not resolve spontaneously over the years with the acquisition of immunological tolerance, but remains often for life (2, 3). Besides, peanut allergy is due to a particularly high number of fatal and near fatal food-related reactions (4).To date, the gold-standard for the diagnosis of food allergies is the double-blind placebo controlled food challenge (DBPCFC). The single-blind is a valid alternative: a recent study has shown a 100% correlation between a positive DBPCFC and a positive single-blind oral food challenge, evaluated in peanut allergy patients. The open oral food challenge (OFC) is the commonest choice for the greater ease of execution, although it is still expensive, time-consuming and potentially dangerous (1).

Skin prick test (SPT) and serum-specific immunoglobulin E (sIgE) dosage are the fisrt steps in the diagnostic work up but they do not always correlate with clinical reactivity: they provide the similar good sensitivity (90%) and low specificity (50%). Besides, sIgE serum values or SPT wheal size cannot accurately predict whether the patient will have a severe reaction and the degree of severity. They express only the likelihood of an IgE-mediated reaction of variable intensity (5). In these previous years, a new diagnostic test, the component-resolved diagnosis (CRD). has come to our aid. It quantifies the concentration of the sIgE to different allergenic components of several allergens including peanut. Among the allergenic components of peanut, we may differentiate 4 groups of proteins: profilines (Ara h 5); pathogenesis-related protein 10 also known as PR-10 (Ara h 8); lipid transfer proteins or LTPs (Ara h 9) and storage proteins (Ara h 1, Ara h 2 and Ara h 3). In relation to their resistance to heat (cooking) and enzymatic digestion (gastric and intestinal enzymes), they have an increasing hazard profile from profilines to storage proteins. Profilines and PR10 are usually observed in mild reactions, like the typical oral allergy syndrome (OAS), and cross-react with pollens. LTPs and storage proteins are gastro-stable and heat-stable, and are often implicated in severe systemic reactions (2).

The aim of the present study was to evaluate the utility of peanut CRD performed before OFC and the differences in peanut component recognition patterns in Italian children with suspected peanut allergy.

## Materials and methods

The children of the study group were selected among patients attending the outpatient clinic of Pediatric Allergy at the University Hospital of Verona from September 2014 to April 2015. 32 patients (n= 23 males, 72%; median age of all patients 9 years, interquartile range (IQR) 6.0-11.0 years) with history of peanut allergy were recruited. Previous allergic reactions to peanut were reported by the children's parents, after a thorough medical history about previous peanut ingestion or exposure. The most common clinical manifestation referred by children parents was urticarial/angioedema (90% of the cases), followed by respiratory symptoms (15% of cases) and gastrointestinal symptoms such as abdominal pain and vomit (6%). To assess peanut sensitation, SPTs were performed in all patients (positive result for wheal size≥ 3mm) (Table 1). A blood sample was collected to evaluate the serum concentration of sIgE to whole peanut and the recombinant allergen components Ara h 1, 2, 3, 8, and 9 with Immuno CAP (Thermo Fisher Scientific, Uppsala, Sweden). All children underwent open oral food challenge (OFC) that was performed according to the recent guidelines on food allergy and anaphylaxis guidelines from the European Academy of Allergy and Clinical Immunology (EAACI) (6). The start dosage was of 6 mg of peanut flour (3 mg of peanut proteins) with subsequent incremental dosages of 20 mg, 60 mg, 200 mg, 600 mg, 2000 mg and 6000 mg, administered with a time interval between two doses of 30 min. The OFC was considered positive after development of at least 2 objective signs: skin rash, sneezing, vomiting, cough, wheeze, and >20% decrease in forced expiratory volume 1 (FEV1). The FEV1 is the volume of air force fully exhaled in 1 second and is the best marker of broncho-spasm. All tests (skin prick test, blood exams and OFC) were performed as part of regular patient management and after obtaining informed consent from the children parents. All data were collected anonymously and all medical procedures were performed according to the code of conduct for medical research approved by the hospital’s medical ethical committee. The statistical analyses were performed using GraphPad Prism software package (version 6, GraphPad Software Inc., San Diego, CA, USA) and the results were considered stastically significant at a p-value less than 0.001. Logistic regression analysis and ROC curve were used to evaluate the laboratory findings.

**Table 1:** Characteristics of the studied population

	**All patients ** **(n= 32)**	**Allergic patients** **(n= 12)**	**Tolerant patients** **(n= 20)**
Age, years, median (IQR)	8.5(6.0-11.0)	6(2.75- 9.5)	10(8.0-11.75)
Sex, male, no. (%)	23(72)	10(83.3)	13(65)
Skin prick test, mm, median (IQR)	4.0(3.0-5.0)	6.5(4.0-8.0)	4.0(2.25-4.0)

**Table 2:** Median values of serum sIgE to whole peanut and peanut components

	**Tolerant**	**Allergic**	**P** ** value**
**Peanut kU**_A_ **/L, median (IQR)**	5.35 (1.44-16.6)	5.81 (0.97-18.07)	NS
**Positive peanut, %** *	17 (85)	11 (91.67)	
**rAra h 1 kU**_A_ **/L, median (IQR)**	0.1 (0.1-0.1)	0.1 (0.1-0.12)	NS
**Positive Ara h 1, %** *	2 (10)	1 (8.33)	
**rAra h 2 kU**_A_ **/L, median (IQR) **	0.1 (0.1-0.12)	0.75 (0.22-4.34)	< 0.001
**Positive Ara h 2, %** *	2 (10)	7 (58.33)	
**rAra h 3 kU**_A_ **/L, median (IQR) **	0.1 (0.1-0.1)	0.1 (0.1-0.15)	NS
**Positive Ara h 3, %** *	0	2 (16.66)	
**rAra h 8 kU**_A_ **/L, median (IQR) **	0.23 (0.1-16.6)	0.13 (0.1-1.9)	NS
**Positive Ara h 8, %** *	8 (40)	3 (25)	
**rAra h 9 kU**_A_ **/L, median (IQR) **	2.23 (0.1-8.52)	0.1 (0.1-0.73)	NS
**Positive Ara h 9, %** *	9 (85)	3 (25)	

* Positive IgE considered value at ≥ 0.35 kUA/L

## Results

After OFC, 12 (37.5%) out of 32 children with reported history of peanut allergy resulted positive and were considered really allergic. The remaining 20 patients were defined as tolerant due to absence of the positive clinical criteria to OFC described in the methods section. Patients with absence of clinically significant reactions to OFC, may be considered not at risk of reactions to subsequent ingestion of peanuts.

Relative to SPTs, no significant difference in wheal size was observed between allergic and tolerant patients (Table 1).

**Fig 1:**
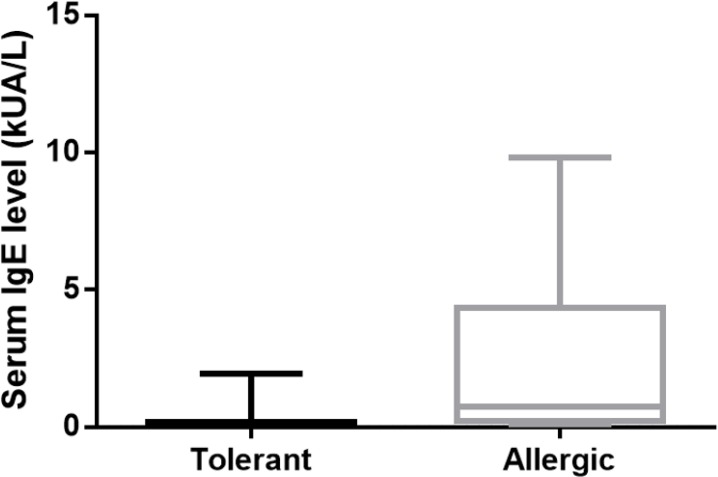
Median values of sIgE to Ara h 2 in allergic and tolerant children to oral peanut challenge

The ImmunoCAP results have not shown significant differences in the serum concentration of sIgE to peanut and the peanut components Ara h 1, 3, 8 and 9 between allergic and tolerant children. Conversely, a statistical significant difference was evident in the sIgE to Ara h 2 between allergic and tolerant patients: 0.75 kUA/l (IQR: 0.22–4.34 kUA/l) in the allergic group versus 0.1 kUA/l (IQR: 0.1–0.12 kUA/l) in the tolerant one (P< 0.001). Table 2*** s***hows the median values and the range of serum sIgE to whole peanut and peanut components in tolerant and allergic patients (Figure 1). Noteworthy, 5 of 20 tolerant children had IgE to peanut> 15 kUA/l (median, 23.5 kUA/l; IQR: 17.05-33.2 kUA/l) which is commonly considered a predictive value of peanut allergy.

## Discussion

The present study adds confirmatory evidence regarding the use of sIgE to Ara h 2 in the diagnostic work up of patients with peanut sensitization and suspected peanut allergy. sIgE to whole peanut and SPT are characterized by a similar sensitivity (90%) and specificity (50%) and are useful in the diagnostic work-up (5, 7), but they can not be used to predict the outcome of the OFC, that remains the gold-standard for food allergy diagnosis although it is expensive, potentially dangerous and time-consuming.

As mentioned previously, Ara h 2 belongs to the family of the storage proteins that, for their gastro- and thermo-stability, are usually considered dangerous because often implicated in severe IgE-mediated reactions.

The present study was performed to evaluate the possible use of CRD in peanut allergy as a test able to predict the outcome of the OFC, and showed that the sIgE to Ara h 2 responds to this requirement. Other studies with similar aim were carried out. Notably, Lieberman et al. carried the dosage of sIgE to peanut and its components (Ara h 1, 2, 3, and 8), and subsequently a DBPCFC to 167 children (7-15 years of age) and showed that sIgE to Ara h 2 dosage was the most specific test for challenge-proven peanut allergy (8). Another recent Asutralian study on 152 children with similar methodology has demonstrated a CRD specificity of 93% (9). Similarly, Nicolaou et al., obtained the high predictive value for clinical reactivity of Ara h 2 sIgE by studying 79 children, using ImmunoCAP method (Phadia, Uppsala, Sweden) which is a routinely available laboratory test, also used in our study. They detected the best cutoff point of 0.35 kUA/L (100% of sensitivity; 96.08% of specificity) (10).

Another cuttoff point was detected by Klemans et al. who enlisted 100 pediatric patients with peanut allergy who have been subjected to OFC and dosage of sIgE to Ara h 1, 2, 3, and 8. A cutoff point of >5kU/L gave the best results (positive predictive value of 96% and negative predictive value of 71%). Using this cutoff, it is possible to predict the DBPCFC outcome in 50% of patients with an accuracy of 100% (11).

An american study evaluated 186 children that were divided in 4 groups: 20 nonatopic controls, 58 asymptomatically peanut-sensitized (PS) children and 108 peanut-allergy (PA) children (55 non-anaphylactic and 53 anaphylactic patients). The dosage of sIgE and sIgG4 to 103 allergens (including 4 peanut allergens: Ara h 1–3 and 8) showed that the sIgE to Ara h 1–3 and Gly m 5–6 (soy allergens) were significantly higher in PA patients than in the asymptomatically sensitized children (P <0.00001), with a similar but less evident trend observed for sIgG4 to Ara h 2 (P<0.01). The best predictors of symptomatic sensitization were sIgE to Ara h 2, with a best cutoff of 0.65 ISU-E, that conferred sensitivity of 99.1% and a specificity of 98.3%, but without the ability to differentiate peanut anaphylaxis from non-anaphylactic PA (12).

Klemans et al. evaluated 37 patients (22 adults and 15 children) with sensitization to peanut and a positive DBPCFC with the aim to compare the use of the peanut components Ara h 1, 2, 3 and 8 with 4 different techniques (i.e. multi- plexed microarray, single- plexed IgE assay, SPT and immunoblot). They found a similar sensitivity between the 4 techniques but in children sIgE to Ara h 2 evaluated through single- plexed assay showed the best sensitivity (100% vs 76.2%) (13). 

A further confirmation of the Ara h 2 sIgE diagnostic importance was given by Koppelmn et al. who carried out the analysis of IgE binding to purified Ara h 2 on immunoblot, SPT and basophil activation test (BAT) in 32 adult peanut-allergic patients after OFC execution. They found that Ara h 2 was identified most frequently in all tests and determined both positive SPT and basophil degranulation at lowest concentrations (14). Other studies add value to these data because they have already shown that BAT has a good sensitivity to detect severe peanut allergies; that a negative basophil allergen threshold sensitivity usually excludes a peanut clinical reactivity; that SPTs are effective to individual recombinant peanut allergens, mostly to Ara h 2 (15-18).

There is a great number of further studies that demonstrate the prevailing value of Ara h 2 sIgE in the diagnostic workup of peanut allergy in children, using OFC to confirm peanut clinical reactivity (3, 19-23) or only the medical history to evaluate the peanut allergy clinical severity (24, 25).

Other studies show a correlation between sIgE to Ara h 2 and clinical severity of peanut allergy, demonstrating a prevalence of Ara h 2 sIgE in patients with more severe reactions (26).

A recent review that analyzed 32 studies (21 in pediatric populations) to assess the diagnostic value of sIgE to peanut components, concluded that sIgE to Ara h 2 has the best diagnostic accuracy and for this it is eligible for its use in daily clinical practice, especially in children (27).

Finally, some studies have evaluated the serum concentration of IgE and IgG4 to peanut allergen components during rush oral immuno-therapy (OIT), showing that Ara h 2 sIgE and sIgG4 characterized the serological response during the treatment in all patients (in particular a sustained Ara h 2 sIgG4 response) (28, 29). Another study speculated the futuristic use of Ara h 2 for a peptide-based immunotherapy, after the evaluation of MHC-class II-based T cell epitope (30).

The only Italian pediatric study conducted with the limit of the absence of OFC to evaluate the real clinical reactivity to peanut, highlighted a prevalence of Ara h 9 (LTP) and Ara h 8 (PR10) sIgE in school age children and adolescents, with an increasing trend with age as probable expression of cross- reactivity with pollens (secondary sensitization). In preschool children Ara h 1 and Ara h 2 (storage proteins) were prevalent (genuine sensitization) (2).

In conclusion, the present study adds confimatory evidence about the central role of sIgE to Ara h 2 in the diagnostic work up of peanut allergy and it has been in line with recent reports in various populations of Northern Europe, US and Australia. In agreement with other authors, we suggest its possible use in daily clinical practice and its utility to avoid dangerous, expensive and time-consuming OFCs.

## Conflict of interest

The authors declared no conflict of interest.
